# Transition to adulthood and adult health care for patients with sickle cell disease or cystic fibrosis: Current practices and research priorities

**DOI:** 10.1017/cts.2018.338

**Published:** 2019-02-05

**Authors:** Sophie Lanzkron, Gregory S. Sawicki, Kathryn L. Hassell, Michael W. Konstan, Robert I. Liem, Susanna A. McColley

**Affiliations:** 1 Department of Medicine, Johns Hopkins School of Medicine, Baltimore, MD, USA; 2 Department of Pediatrics, Harvard Medical School, Boston, MA, USA; 3 Department of Medicine, Division of Hematology, Colorado Sickle Cell Treatment and Research Center, University of Colorado, Aurora, CO, USA; 4 Department of Pediatrics, Case Western Reserve University School of Medicine, Cleveland, OH, USA; 5 Department of Pediatrics, Northwestern University Feinberg School of Medicine, Chicago, IL, USA

**Keywords:** Healthcare transition, adolescence, young adulthood, cystic fibrosis, sickle cell disease.

## Abstract

**Introduction:**

A growing population of adults living with severe, chronic childhood-onset health conditions has created a need for specialized health care delivered by providers who have expertise both in adult medicine and in those conditions. Optimal care of these patients requires systematic approaches to healthcare transition (HCT). Guidelines for HCT exist, but gaps in care occur, and there are limited data on outcomes of HCT processes.

**Methods:**

The Single Disease Workgroup of the Lifespan Domain Task Force of the National Center for Advancing Translational Sciences Clinical and Translational Science Award programs convened a group to review the current state of HCT and to identify gaps in research and practice. Using cystic fibrosis and sickle cell disease as models, key themes were developed. A literature search identified general and disease-specific articles. We summarized key findings.

**Results:**

We identified literature characterizing patient, parent and healthcare provider perspectives, recommendations for transition care, and barriers to effective transition.

**Conclusions:**

With increased survival of patients with severe childhood onset diseases, ongoing study of effective transition practices is essential as survival increases for severe childhood onset diseases. We propose pragmatic methods to enhance transition research to improve health and key outcomes.

## Introduction

Treatment advances for diseases that were previously fatal in childhood have resulted in a growing population of adults living with severe, chronic childhood-onset health conditions. Adults with these conditions often receive care from pediatric providers longer than their healthy peers. However, psychosocial and medical needs, including the need for adult-focused preventive services, co-morbidities related to and separate from child-onset conditions, and other care needs including contraceptive and reproductive care, mandates entry into a system focused on adult health care. There is thus a compelling need for specialized health care delivered by providers who have expertise in both adult medicine and in chronic conditions that begin in childhood. Because of significant physical, psychological, social, vocational, and other challenges of surviving to adulthood with chronic health conditions, the process of navigation from the pediatric to adult healthcare environment is complex. This process, termed health care transition (HCT), is defined as “a purposeful, planned process that supports adolescents and young adults with chronic health conditions and disabilities to move from child-centered to adult-oriented health care practices, providers, programs, and facilities” [1]. HCT includes, but is not limited to, transfer of care, defined as movement of a patient from one healthcare provider or facility to another, along with medical records and other information. The rationale for HCT, and for transition programs to assess and promote readiness for adult care, is to optimize health and social outcomes not only during late adolescence and young adulthood, but also during the entire lifespan. The critical need for HCT processes and programs to promote optimal health outcomes for adult survivors of previously fatal childhood disorders was noted in an Institute of Medicine (now The National Academy of Medicine) report on the “Clinical and Translational Science Awardees funded by the National Center for Advancing Translational Sciences” [2].

Clinical guidelines for HCT include recommendations for routine use of structured transition readiness assessments and development of transition care plans [3]. As HCT processes and programs have been developed, the growing literature base has focused on assessment, processes, patient and provider experiences, and outcomes [4, 5]. Despite these and other efforts to improve HCT over the past decade, national surveys of parents and youth with chronic health conditions have repeatedly shown that HCT planning is often lacking [6–8]. Deficiencies in HCT processes are likely to have greatest impact on health outcomes in populations with progressive disease. As a consequence, the Single Disease Workgroup of the CTSA Lifespan Task Force conducted a study of HCT in severe, progressive childhood-onset conditions. Sickle cell disease (SCD) and cystic fibrosis (CF) were chosen for study as they are well-characterized disorders caused by homozygosity of mutations in a single gene, are progressive, and have been the subject of many reports on HCT.

The aims of this report are to provide an introduction to SCD and CF as the specific disorders evaluated by the Workgroup, present essential components of successful HCT and barriers to them, and propose research priorities and methods for further study of HCT.

## Methods

A literature search was performed via PubMed utilizing the search terms (“continuity of patient care”[Mesh] OR “pediatric transition to adult care”[tw] OR “pediatric transition”[tw] OR ((“pediatric”[tw] OR “child*”[tw] OR “adolescent*”[tw] OR “adolescence”[tw] OR “teen*”[tw] OR “young adult”[tw] OR “juvenile*”[tw] OR “youth”[tw]) AND (“transition*”[tw] OR “transfer*”[tw] OR “handoff*”[tw] OR “handover*”[tw] OR “hand-over”[tw] OR “HCT”[tw] OR “transition planning”[tw] OR “transition protocol”[tw] OR “clinical pathway”[tw] OR “self-efficacy”[tw] OR “self efficacy”[tw] OR “self-care”[tw] OR “self care”[tw]) AND “adult*”[tw])) AND (“cystic fibrosis”[Mesh] OR “cystic fibrosis”[tw] OR “mucoviscidosis”[tw] OR “CF”[tw] OR “sickle cell”[tw]). The authors utilized the search and other literature to evaluate current transition knowledge and practice and identify gaps in current care and research.

## Epidemiology and Treatment of Sickle Cell Disease

SCD is one of the most common inherited blood disorders in individuals of African, Hispanic, Middle-Eastern, and Asian Indian descent, affecting more than 100,000 individuals in the US [9, 10]. Of these, 60%–70% have the most severe form of the disease, sickle cell anemia or Hemoglobin SS (HbSS). Due to the presence of an atypical form of hemoglobin, red blood cells take on a “sickle” shape and cause injury and intermittent occlusion of the microvasculature, resulting in early damage and loss of function of several organs, including the spleen. Manifestations, including fatal sepsis, can occur as early as 2 months of life due to early splenic injury; historically, sickle cell anemia led to significant early childhood mortality before the age of 5 years. The finding that administration of prophylactic penicillin could successfully prevent this complication prompted universal newborn screening for sickle cell anemia in the US [11]. This intervention, along with comprehensive vaccination and parental education regarding early childhood complications, has nearly eliminated the early risk of mortality [9, 12]. Early to middle childhood is characterized by potentially life-threatening complications, including stroke and/or the onset of clinically silent cerebral ischemia, which can compromise neurocognitive development and function. Significant reduction in neurological complications can be achieved through identification of children at risk using transcranial Doppler and neuroimaging and subsequent life-long chronic transfusion therapy or use of hydroxyurea, an oral agent that induces the production of fetal hemoglobin, reducing the potential for sickling of the red blood cell [12, 13]. Episodes of severe acute pain (called “crisis”) that require hospitalization and potent analgesics may begin in early childhood and occur throughout life. Crises are not predictable but can be triggered by factors including dehydration, exposure to extremes of temperature, or other stressors. Some of these episodes lead to severe sickling in the lung, called “acute chest syndrome,” a common acute cause of mortality in individuals with SCD [14, 15]. The frequency of these acute severe complications may be reduced by the use of hydroxyurea.

Advances in care made over the last 30 years have markedly reduced childhood mortality in SCD to <10%, and it is currently estimated that at least 50% of the sickle cell population are adults [16]. Despite reduction in childhood mortality, available data suggest little improvement in the estimated mean age of death of 40–45 years for those with HbSS, while a subset of severely affected individuals die in their 20s or 30s [17]. The course of disease in adolescence and young adulthood is associated with cumulative chronic organ damage, particularly in the lungs and kidneys, and can occur even with adherence to disease-modifying therapies [18]. Avascular necrosis of the hips and shoulders and retinopathy create additional morbidity. During adolescence and young adulthood, chronic pain emerges for a significant subset of those most severely affected, superimposing additional burden [19]. As SCD disproportionately affects populations with historically lower socioeconomic status, the majority of children and adults with the disease rely on healthcare access through Medicaid or other government-based health insurance programs [20].

The course of SCD is further worsened if monitoring and early interventions for these complications and therapies such as hydroxyurea and transfusion are interrupted. Unfortunately, adolescents and young adults may be less likely to engage in healthy behaviors, including adherence to daily medication or monthly clinic visits for transfusion therapy. Feeling well, some may opt out of interventions, with severe clinical consequences [21]. Available data demonstrate an association between a decrease in the use of therapies and an increase in SCD activity and emergency room visits beginning in mid-adolescence [18, 22].

## Epidemiology and Treatment of Cystic Fibrosis

CF is the most common lethal genetic disease in Caucasians, occurring in 1 in 3000 live births [23]. African Americans and individuals from other racial and ethnic groups are affected less frequently, but as demographics change in the US, the prevalence of CF in minorities is increasing, particularly among Hispanic Americans. In 2016, 29,497 CF patients were reported to the Cystic Fibrosis Foundation (CFF) Patient Registry; it is estimated that they represent 84% of CF patients in the US [24]. The disease is caused by absent or defective CF transmembrane conductance regulator protein, which regulates salt and water transport across cells. Many organ systems in the body are affected by CF, but its effects on the lungs and digestive tract are most pronounced. Thick secretions obstruct the airways, and chronic bacterial infections and inflammation slowly destroy the lungs. Most people with CF have pancreatic insufficiency, which leads to poor growth and nutritional status. Other clinical manifestations of CF include infertility in most males [23].

Untreated, CF is usually fatal in early childhood. Therapies aimed at treating the lung and digestive abnormalities, along with a comprehensive team-based approach to evaluation and treatment, has led to a marked improvement in survival. Median predicted survival has more than doubled since the mid-1980s, and reached an average of 42.7 years in the time period 2012‒2016 [24].

Despite the increase in life expectancy, the median age of the CF population is 19 years, and the median age at death is 29.6 years [24]. A cluster of deaths begins during late adolescence that peaks in the early 20s, primarily due to an accelerated loss of lung function that begins in early adolescence. Among many factors that contribute to loss of lung function, epidemiologic studies have identified an increase in the frequency of pulmonary exacerbations as a prime factor, particularly among adolescents [25]. Pulmonary exacerbations are characterized by an increase in respiratory symptoms such as cough and sputum and an acute drop in lung function [26]. Even with aggressive treatment of these events, which often require hospitalization for intravenous antibiotics for 10–14 days, in many cases pulmonary function does not fully recover [27]. Moreover, exacerbations result in missed school or work, both of which markedly interfere with a teenager’s or young adult’s ability to keep up with their peer group. A proportion of CF patients experience a very rapid drop in pulmonary function (>10% predicted forced expiratory volume in 1 s) between 18 and 22 years of age; higher forced expiratory volume in 1 s and lower body mass index at age 18 are risk factors for this rapid decline [28].

Maintaining lung health and nutritional status are paramount to the well-being of people with CF, and a complex chronic treatment regimen is recommended to prevent or delay the occurrence of exacerbations [29]. It is not uncommon for a person with CF, even at the earliest stages of lung disease, to spend more than 1–2 hours per day taking oral and inhaled medications and performing airway clearance therapy [30]. The proportion of patients prescribed these therapies is greatest during late adolescence and early adulthood [24], a challenging period for adherence.

Treatment is expensive, including newer gene mutation-specific CF transmembrane conductance regulator modulator therapies that cost nearly $300,000 per year [31]. A large proportion of children and some adults with CF are insured under Medicaid or other state programs [24] that may not provide coverage for CF therapies recommended by published guidelines. Adults who meet federal disability criteria may be insured by Medicare, which, in some cases, has high out-of-pocket costs for medications. Commercial insurance gaps have lessened under the Affordable Care Act, since young adults can stay on their parents’ health insurance plan until age 26, and cannot be denied insurance due to a preexisting condition. Although private insurance provides access to CF care, it does not necessarily guarantee better health, and lack of any insurance is associated with poor utilization and worse health outcomes [32]. Similar to other chronic health conditions, lower socioeconomic status is associated with poorer health outcomes in CF, as is race and ethnic minority status, even after adjusting for socioeconomic status [33–36].

Having any chronic disease is stressful, and CF is no exception. Depression and anxiety disorders among people with CF increases during adolescence [24, 37]. There is now an increased emphasis on identifying and treating mental health issues in patients with CF, including a consensus statement on screening for and treating depression and anxiety [38]. Risky behaviors that lead to poor health, including tobacco, alcohol, and substance abuse, also occur in individuals with CF, but less frequently than in the general young adult population [39].

With increasing survival and an improved outlook for adults with CF, many are pursuing higher education and employment. About two-thirds of adults with CF are either students or are working, and many are in committed relationships and having children of their own [24]. Helping adolescents transition successfully to adulthood is thus critically important not only to their quality of life, but also for their future personal and professional success.

## Transition Issues in Sickle Cell Disease and Cystic Fibrosis

Adolescence is a precarious time for patients with either SCD or CF, as it marks an intersection of normal developmental challenges superimposed on a period of disease progression. In SCD, the frequency of pain crises increases in late adolescence. In CF, lung function decline accelerates during adolescence, and despite more aggressive management and new therapies, the onset of more rapid decline has shifted minimally from around 10 years of age to 14 years. In both disorders, the need to maintain prescribed regimens of medication, lifestyle interventions such as nutrition and hydration, and attend health care visits increase in adolescence and young adulthood—the very time of life when patients are most vulnerable. It is in this setting of heightened physical and psychosocial risk that transfer of care should be initiated.

HCT processes ultimately need to create a bridge between pediatric and adult-focused health systems. The six core elements of HCT have most recently been described as a framework for creating an HCT program. They are: develop a transition policy, maintain a system of tracking, assess transition readiness, engage in active transition planning, create a system for transfer of care, and assess transfer completion [40].

The CFF began advocating for the development and accreditation of distinct adult CF programs in the late 1990s and, by 2000, CF care centers providing care for more than 40 individuals older than age 21 were mandated to create and sustain a dedicated adult CF program. Despite these recommendations, a survey of US CF programs in 2008 revealed significant variability in transition support provided to their patient populations [41]. In contrast, there is no accrediting organization that requires a specific structure of HCT for SCD, and the most recent set of management recommendations from the National Heart Lung and Blood Institute do not provide guidance regarding transition [42].

In addition to lack of consistent practices around HCT, numerous barriers to successful HCT exist for patients and their caregivers, as well as health care providers and systems (Table 1). Patient and caregiver barriers include comorbidities that can make transition to independence more difficult, notably mental and behavioral health and cognitive and educational challenges. Poor understanding of current care and its benefits, with reliance on caregivers to organize and provide care, is an important barrier, as is attachment to a long-term pediatric care team and perception of lack of knowledge and experience. Both may result in resistance to initiating and completing transition processes. When adult CF programs were first established more than two decades ago, several studies documented concerns voiced by pediatric CF providers, patients, and family members about the ability of adult staff to meet patients’ medical needs [43–45]. One study documented reluctance among adolescents and adults with CF to transfer care due to concerns over whether they could establish strong relationships with CF physicians in an adult program [46]. Similarly, a mistrust of adult physicians was indicated by SCD patients, adding to the stress of finding/maintaining insurance, working with employers, and the impact of the condition on personal relationships [47]. These concerns, combined with the timing of transition during a vulnerable time in the trajectory of disease in both disorders, put further pressure on the HCT process. Health system barriers include inadequate training, experience and access for adult care practitioners, resistance by pediatric practitioners, lack of coordination and shared systems, and differences in payers and payment structures.

**Table 1:** Barriers to transition in sickle cell disease and cystic fibrosis

Youth/caregiver barriers
Depression, anxiety, other mental health disorders and behavioral abnormalities
Academic failure due to lost school days
Cognitive deficits
Perceived lack of disease knowledge by practitioners
Youth lack of understanding of current therapy and its benefits; poor adherence attachment to pediatric care team (youth and/or parent)
Psychosocial resistance (youth and/or parent)
Health system barriers
Inadequate training of primary and subspecialty care providers
Shortage of mental health practitioners and services
Lack of appropriate training on treating childhood-onset health conditions among adult-focused health care providers
Resistance of pediatric health care teams to initiate transition activities and transfer to adult systems
Challenges in care coordination across systems (lack of integration of health records)
Poor access to adequate health insurance coverage in young adulthood; high proportion of populations receiving public insurance through Medicaid

### Barriers to Successful Transition: Mental Health

Children and adults with chronic health conditions are known to have higher levels of depression and anxiety [48]. In CF, the prevalence of depression in adolescents and adults has been reported to be 10% and 19%, respectively, while 22% and 32% of adolescents and adults screen positive for anxiety [24]. Psychological distress in CF is associated with decreased health-related quality of life, decreased adherence to prescribed therapies, decreased pulmonary function, increased hospitalization, and increased health care costs. Published studies examining depression in SCD report prevalence rates as high as 57% [49]. Depression has been associated with higher health care utilization [49] and decreased adherence to disease-modifying therapy [50] in individuals with SCD. Compounding mental health issues are concerns about substance abuse in adolescents. In a study comparing adolescents with CF or SCD with their healthy peers, the chronically ill teens reported significantly less use of tobacco, marijuana, alcohol, cocaine, and injection drug use. However, a recent study found that 21% of the teens with CF and 30% of those with SCD had smoked, suggesting that screening for risky behaviors is needed [51]. While there are no studies directly examining the role of psychiatric comorbidities on the success of transition in SCD or CF, the potential for psychiatric comorbidities to hinder adherence to appointments and effective disease self-management can be an additional barrier to successful transition. Assessing for and implementing interventions to address mental health during adolescence and early adulthood can be important steps to improving transition.

### Barriers to Successful Transition: Adherence

Numerous studies have evaluated adherence to recommended therapies and routine monitoring visits and procedures in adolescents with CF and SCD. Adherence rates in CF are clearly suboptimal, and the lowest rates of adherence in CF are among adolescents and young adults [52]. Though highly complex, a number of themes around nonadherence have emerged in CF. These include the role of recursive perception—how an individual perceives how others view them in turn influences individuals to act in a manner that aligns with how they want to be seen. For example, if an individual does not wish to be viewed as ill, then they will not take medication, which is viewed as being only for those who are ill. Other themes that have been identified in CF include: inadequate knowledge and lack of understanding of the benefits of medication; psychosocial resistance, which includes struggles with parents, psychological responses, and denial; and finally, educated nonadherence, that is, when a patient makes their own decisions based on what is most important to them despite understanding their physician’s advice [53, 54]. Similar concerns over nonadherence exist for patients with SCD, though less is known about what drives nonadherence in adolescents with SCD. Clinicians who treat patients with SCD report that nonadherence to medications and lab monitoring are barriers to prescribing hydroxyurea in SCD. However, clinicians’ perceptions of the important factors that lead to nonadherence do not always align with factors that patients identify as barriers to adherence. In a systematic review of patients with SCD, adherence rates ranged from 16% to 89%. Patient-identified barriers included fear of side effects, incorrect dosing, and forgetting to take medications or attend clinic. Importantly, nonadherence was associated with more vaso-occlusive crises and hospitalizations [55]. Overall, transition interventions must address barriers to adherence and appropriate self-management in order to achieve optimal health outcomes during HCTs and into adulthood.

### Barriers to Successful Transition: Education/Employment

During focus groups, young adults with SCD define a successful transition as one where they are able to function as an independent member of society, manage their health, and generate income [56]. While patients recognize what is needed, they are often poorly prepared for transition to adult care [57]. Interest in learning about the process of transition increases as adolescents age, but interest is lower in those with more severe disease [58]. Similar findings have been seen in adolescents with CF, where patients have been noted to have poor knowledge about lung disease and even lower scores in nutrition knowledge [54]. In the same study, investigators found that lower scores on disease knowledge assessments were associated with lower adherence to recommended therapy.

Improving knowledge and the ability to navigate the health care system may be particularly challenging for some young adults living with SCD, as cognitive impairment due to brain injury and/or social and environmental disadvantage may play a role and are less amenable to intervention [58]. Both overt stroke and silent cerebral infarcts contribute to significant neurocognitive dysfunction in SCD. When comparing children with SCD to their unaffected siblings, those with SCD have lower global IQ scores [58]. Children with sickle cerebral infarct also have a much higher rate of grade failure than their sibling controls [59]. Even those children without cerebral infarct are at risk for poor education attainment due to many missed days of school from painful events [59]. Effective interventions to overcome educational barriers in adolescents with CF or SCD have yet to be identified. One study that tested a generic 2-day educational program for young adults with CF, diabetes, and inflammatory bowel disease showed an improvement in self-efficacy for those with diabetes and inflammatory bowel disease but no improvement for those with CF [60].

### Barriers to Transition: Primary Care

Across the population, adolescents and young adults have fewer primary care visits than younger children or older adults [40, 61]. Furthermore, youth with serious chronic health conditions often view their subspecialty physician and disease-focused ancillary care providers as their “medical home.” This may lead to discontinuity of preventive services and inadequate coordination of care during transition from pediatric to adult healthcare settings. At the health system level, training of internists and other primary care physicians in the care of childhood-onset chronic conditions is also lacking and may lead to challenges in access to appropriate care for certain young adults. For example, in a survey of over 500 internists, only 15% and 32% reported feeling comfortable being a primary care physician for a patient with CF or SCD, respectively [62]. In a similar survey for internal medicine trainees, <25% reported comfort with outpatient management of CF [63].

## Research Priorities

### Defining Outcomes of Successful Health Care Transition: Measurement and Standardization

In general, there is a scarcity of published evidence supporting which HCT practices are associated with improved intermediate to long-term health and functional outcomes in adulthood. Before developing an evidence base for best transition practices, however, appropriate outcome measures must be defined or developed. Outcomes for transition need to be evaluated across a long period of time, and should include measures for transition preparation, transfer of care, and intake and care in an adult-focused health system. Furthermore, it is essential to define desired transition outcomes broadly, not just as discrete markers of disease activity or outcomes, though these should be included when there is clear evidence of their impact on symptoms, survival, health utilization, and other key indicators of health. Examples of measures, including health status, patient reported outcomes, healthcare utilization, and transition measures are summarized in for SCD [64–68] and CF [69, 70] in Table 2.

**Table 2:** Health care transition outcome measure categories and sample measures

Health status	General and specific patient-reported outcomes	Healthcare utilization	Transition measures
Pulmonary function change per year (CF) Body mass index (CF) Pulmonary exacerbation rate per year (CF) PhenX Toolkit Measures (SCD) [64]	Patient-reported outcomes measurement information system Cystic Fibrosis Questionnaire-Revised (CF) [69] Adult Sickle Cell Quality of Life Measurement System [65] Educational attainment Employment status	Use of preventive services Visits to primary care provider Visits to subspeciality provider Emergency department use Hospitalization	TRAQ [70] TIP-RFT [66] KKIS-SCD [67] STARx 9 [68]

CF, cystic fibrosis; KKIS-SCD, Kennedy Krieger Independence Scales-Sickle Cell Disease; SCD, sickle cell disease; TIP-RFT, Transition Intervention Program Readiness for Transition; TRAQ, Transition Readiness Assessment Questionnaire.

Key indicators of health status in CF include lung function, nutritional status, and pulmonary exacerbation rates; and in SCD, include acute care episodes and health care utilization that mirror pain crises and vascular events. In both disorders, adherence to specialist visits is essential for disease management and therapeutic recommendations. Available evidence suggests that transfer to adult care is not associated with worse clinical health outcomes in CF. A retrospective analysis of the CFF Patient Registry using data from 1997 to 2007 found that patients who transferred care from pediatric to adult CF care had less rapid decline in lung function compared with a matched cohort of patients who remained in pediatric care [71]. A more recent CFF Patient Registry analysis showed that gaps in care during transfer to adult CF programs were infrequent and identified risk factors for prolonged gaps in care that could help pediatric programs identify higher risk populations [72]. For those with SCD, an increase in reliance on emergency department care has been noted for individuals in the 15–22-year-old age group, suggesting a lack of access to outpatient care [22]. This may indicate a lack of available providers, lack of adherence to follow-up appointments, or other patient, family, and healthcare system factors. Further research is clearly needed on clinical outcomes in the setting of adequate transition resources for this patient population. This research will require collaboration between health care systems that care for pediatric and adult patients, and could include prospective cohort studies, patient and family data entry into registries or other databases and use of large data sets across several institutions and providers.

A Delphi process of HCT experts classified high priority outcome categories for evaluation of HCT practices as individual patient-reported outcomes, health services outcomes, and social functioning outcomes [73], and similar outcomes were proposed for SCD [74]. Patient-reported outcomes for HCT include quality of life [65], satisfaction with care and patient experience, transition skills and readiness assessments, adherence to therapies, and health condition and self-management knowledge and skills. Several transition readiness assessments have been validated, including the Transition Readiness Assessment Questionnaire, and have been used in evaluating transition preparation programs [70]. An adolescent-reported patient experience measure on transition preparation, the Adolescent Assessment of Preparation for Transition (ADAPT) survey, has been developed and tested in a Medicaid population, and can serve as a measure of health system performance from the youth perspective [5]. Health system outcomes include cost of care, access to care, gaps and fragmentation of care, and other measures of care coordination. Utilization of primary and subspecialty ambulatory services and recommended preventive health or monitoring services may also be important in evaluating outcomes. Overall, research linking patient reported outcomes, health system outcomes, and clinical outcomes during HCT is needed for a holistic view of practices that support individual, healthcare system, and societal benefit.

### Developing Key Research Questions

Research into HCT practices and outcomes must ultimately guide clinicians, health care systems, and policy makers towards practices that are associated with optimal health and social functioning for people with chronic illness through adolescence and well into adulthood. Because transition to adult life occurs over a period of years, generating hypotheses regarding best transition practices must take into account the roles of family, community, and educational systems in addition to those related to the healthcare system per se. Key research questions should also be developed and/or evaluated by stakeholders, including youth anticipating transition, adults who have transitioned from pediatric to adult health care systems, their families, and health care practitioners. This engagement should include validation of existing, and exploration of new, outcome measures that address the highest priorities of patients and their families. For example, while parents and guardians are strongly affected by childhood chronic illness, their contributions to transition processes and outcomes have not been evaluated systematically. Surveys, focus groups, and inclusion of diverse stakeholders in study designs are methods to assure that research findings are most relevant to affected people and populations.

### Research Approaches to Evaluate Health Care Transitions

Retrospective studies of HCT outcomes in CF and SCD have highlighted current successes, barriers, and deficiencies in transition; however, they are inadequate for evaluation of best transition practices across a variety of health care settings. A recent comprehensive review on methods to study service innovations in healthcare and public health reviews approaches and challenges that are widely applicable to transition care [75]. Proposed priorities and methodologies for evaluating HCT are presented in Table 3. Prospective studies evaluating the impact of specific HCT strategies or programs on outcomes over time are essential as affected individuals, their families, and their providers seek strategies to support optimal long-term outcomes. While prospective cohort studies could be designed, they will require long follow-up times and are costly. Furthermore, retention may be especially difficult in adolescent and young adult populations. Utilization of administrative data or electronic health records to determine health trajectories may provide useful information, but require data sharing between organizations and lack details of organizational practices that may influence outcomes. Furthermore, while data sharing through networks is increasing, barriers remain.

**Table 3:** Priorities and methodologies to address transition gaps. Priorities are not ranked. Recommended methodologies are in italics

Priority	Methodologies	Comments
Validate and expand outcome measures through stakeholder engagement	*Surveys* *Focus groups* *Stakeholder involvement in study design*	A combination of condition-specific and generalizable outcome measures may be most useful to drive healthcare system changes
Measure health and social impact of transition services and strategies	Prospective cohort Electronic Health Record data collection across sites of care *Registries including patient reported outcomes*	Methodology should be driven by type and time course of outcome. Pragmatic and cost considerations are barriers to some study designs.
Measure the health and social impact of novel transition interventions	Randomized trials Pragmatic trials *Comparative effectiveness studies* *Quality improvement approaches*	Embedding interventions in ongoing observational studies increases understanding of impact

The impact of one or more specific interventions could be evaluated through randomized trials. However, trials of specific HCT interventions may be unfeasible in rare disease populations and may require very large sample sizes given the variation in outcomes caused by many health and social factors independent of the transition processes themselves. Pragmatic clinical trials may be suitable for the study of transition care but are less feasible for patients who are seen within both primary care and subspecialty practices and have high utilization of acute care hospitals. Given the disadvantages posed by these study designs toward understanding what HCT practices are associated with optimal outcomes, we propose that comparative effectiveness studies, registries that include patient-reported outcomes [76, 77], and quality improvement approaches utilizing available and novel tools and strategies are feasible and amenable to rapid translation into practice. Two comparative effectiveness research studies were funded for SCD in 2017–2024 by the Patient-Centered Outcomes Research Institute, comparing two interventions that have proven effective in facilitating HCT for people with other conditions (https://www.pcori.org/research). One study will assess the benefits of adding peer support to an education program for disease self-management and the other will compare community health worker engagement with patients, use of mobile apps to help individuals manage their condition, and enhanced usual care. Both studies will evaluate quality of life and emergency room/hospital utilization.

Beyond these ongoing studies in SCD, comparative effectiveness research that adequately defines care processes would provide substantial generalizable findings. Transition care could be studied during clinical encounters across sites of care, embedded in a learning health system in which electronic health record, patient-reported outcomes, and other sources of data such as educational attainment and employment are evaluated in the context of specific transition structures and activities. HCT interventions that could be tested in such a framework include the impact of transition care coordinators within a practice, the use of technology-based tools to facilitate acquisition of disease knowledge and skills and reduce barriers to transition, and the impact of dedicated interventions to improve health care delivery such as telemedicine.

Rigorous quality improvement initiatives using multi-site collaborative learning methods would allow for more rapid improvement and generalizability [78], with findings disseminated beyond the collaborative sites when effectiveness is established. In CF, a transition-specific quality improvement program, CF Responsibility, Independence, Self-Care, Education (CF RISE) [79], has been introduced to CF care centers, and implementation studies of the program are ongoing. A common necessity of any of these approaches will be collaboration between pediatric and adult-serving health care providers and institutions. Optimizing clinical and legal interactions between these entities will be essential if we are to address the critical issues impacting long-term care of survivors of childhood-onset diseases. A recent clinical report by the American Academy of Pediatrics, The American Academy of Family Physicians, and the American College of Physicians [80] emphasizes practice-based quality improvement guidance on transition planning, transfer and integration into adult care for youth with or without special health care needs and calls for a stronger evidence base for HCTs.

In summary, while substantial literature supports the need for and utility of systematic approaches to HCT to assure that survivors of severe childhood-onset health conditions have optimal health and psychosocial outcomes, there are significant gaps in knowledge that must be closed before patients, families, and healthcare teams align to optimize services and processes. While laudable efforts are underway to address these gaps, further research developed with patient and family input is essential to assure that best outcomes are both defined and enabled. Comparative effectiveness studies and quality improvement approaches are likely the most feasible research methods given the complexity of processes and the multi-year time frame required to evaluate impact.
